# Integrating Circulating Tumor DNA (ctDNA) Into Postoperative Surveillance After the Resection of Intrahepatic Cholangiocarcinoma: A Proposed Hybrid Imaging-Molecular Framework

**DOI:** 10.7759/cureus.103966

**Published:** 2026-02-20

**Authors:** Mihira Mundhava, Pankaj Jangid, Khushbu Jangir, Niteesh Kumar Yadav, Bhavin Vadodariya, Akshar Patel

**Affiliations:** 1 Medicine, Kyiv Medical University, Kyiv, UKR; 2 Medicine and Surgery, Kyiv Medical University, Kyiv, UKR; 3 Nursing, All India Institute of Medical Sciences, Jodhpur, Jodhpur, IND; 4 Surgery, Kyiv Medical University, Kyiv, UKR; 5 Surgical Oncology, Specialty Surgical Oncology (SSO) Cancer Hospital, Ahmedabad, IND; 6 Oncology, Specialty Surgical Oncology (SSO) Cancer Hospital, Ahmedabad, IND

**Keywords:** cancer recurrence, circulating tumor dna, intrahepatic cholangiocarcinoma, liquid biopsy, minimal residual disease, molecular monitoring, postoperative surveillance

## Abstract

Recurrence after curative-intent resection remains common in intrahepatic cholangiocarcinoma (ICC) and continues to limit long-term survival. Although repeat liver resection may benefit carefully selected patients, this opportunity is often lost because recurrence is detected only after radiographic progression. Postoperative surveillance relies primarily on cross-sectional imaging, which performs well for macroscopic disease but lacks sensitivity for microscopic residual tumor. Consequently, relapse is frequently recognized only after structural visibility, when tumor biology may already be unfavorable. Minimal residual disease (MRD) represents the persistence of viable malignant cells below the threshold of radiographic detection and is increasingly implicated in early relapse. Circulating tumor DNA (ctDNA) analysis enables the detection of tumor-specific genomic alterations in peripheral blood and reflects active tumor biology rather than delayed anatomical change. Across solid tumors, ctDNA positivity has been associated with recurrence lead times of approximately 2-6 months before radiographic detection; however, ICC-specific prospective performance metrics, including sensitivity, specificity, and predictive values, remain limited and incompletely defined. Important practical challenges include assay variability, tumor shedding heterogeneity in biliary tract cancers, clonal hematopoiesis-related false positives, and uncertainty in managing isolated low-level molecular positivity. Accordingly, ctDNA should be considered a complementary rather than a replacement modality. This narrative review synthesizes current imaging and molecular evidence and proposes a hypothesis-generating hybrid imaging-molecular surveillance framework intended to guide future prospective validation rather than serve as an evidence-validated clinical algorithm.

## Introduction and background

In intrahepatic cholangiocarcinoma (ICC), postoperative recurrence following curative-intent liver resection remains common and represents a major obstacle to sustained disease control. Emerging evidence from international multicenter analyses indicates that repeat liver resection may offer meaningful clinical benefit in carefully selected patients with recurrent disease. In recent series, carefully selected patients undergoing repeat resection for recurrent ICC achieved a reported five-year overall survival of approximately 34% [1]. Minimal residual disease (MRD), also referred to as measurable residual disease, signifies the persistence of a small population of cancer cells following treatment [2-7]. In hematologic malignancies, MRD reflects persistent circulating disease within a systemic compartment. In contrast, solid tumors such as ICC demonstrate spatially heterogeneous growth patterns, and MRD represents occult micrometastatic deposits or minimal residual tumor burden following resection rather than sustained circulating tumor cell (CTC) populations [2-7]. Accordingly, application of hematologic MRD paradigms to solid tumors requires biological reinterpretation rather than direct extrapolation [2-7]. Despite its conceptual appeal, MRD assessment in solid tumors presents important limitations. Tumor evolution under therapeutic pressure may alter mutational profiles, and biological confounders such as clonal hematopoiesis of indeterminate potential can generate false-positive signals [4-6]. Reliable interpretation therefore requires validated assays, appropriate analytical thresholds, and careful clinical correlation [4-6]. Current postoperative surveillance relies predominantly on cross-sectional imaging modalities such as computed tomography (CT) and magnetic resonance imaging (MRI) [4,6]. While indispensable for diagnosis and staging, imaging is inherently limited to detecting macroscopic structural abnormalities and lacks sensitivity for microscopic residual disease [4,6]. As a result, recurrence is often identified only after radiographic progression becomes evident. Molecular approaches such as circulating tumor DNA (ctDNA) analysis offer the potential to detect tumor-specific genomic alterations in peripheral blood before anatomical progression is visible [4-6]. Across solid tumors, postoperative ctDNA positivity has been associated with increased recurrence risk and earlier detection of relapse; however, disease-specific performance characteristics vary among tumor types [4]. These molecular signals reflect biologically active residual disease and provide a complementary dimension of surveillance beyond structural imaging.

Although imaging remains indispensable for the diagnosis, staging, and assessment of established disease, it is inherently limited to the detection of macroscopic structural abnormalities and may not reliably identify microscopic residual disease [4]. In contrast, molecular assays such as liquid biopsy provide the potential to detect tumor-specific genomic alterations before anatomical progression becomes radiographically apparent [4-6]. Earlier molecular identification of recurrence may allow for closer monitoring or earlier therapeutic consideration; however, prospective validation is required to determine whether such integration translates into improved survival outcomes [4-6]. Practical challenges, including assay standardization, cost, and interpretation of biologic confounders, currently limit routine implementation in ICC [4-6]. In this review, imaging-based surveillance and liquid biopsy approaches are examined in parallel to evaluate their relative strengths and limitations and to explore whether an integrated strategy may help preserve opportunities for curative-intent repeat resection.

## Review

Limitations of traditional imaging

Spatial Resolution Limits

Conventional imaging modalities, including CT and MRI, remain central to the staging and surveillance of ICC, yet their ability to detect very small lesions is inherently limited [8]. Although much high-resolution imaging data in liver malignancies derives from hepatocellular carcinoma (HCC) cohorts, the same physical constraints of image resolution apply to ICC, particularly in the postoperative setting where residual disease may be microscopic and radiologically inapparent [9-11]. A fundamental limitation of CT and MRI is spatial resolution. Malignant cell clusters measuring only a few millimeters may fall below detection thresholds. While advances in liver imaging have improved lesion characterization, accurately identifying lesions smaller than 10 mm remains challenging [8]. For example, diffusion-weighted MRI has demonstrated limited sensitivity for very small hepatic lesions in HCC cohorts [12]. Similarly, positron emission tomography (PET) is restricted by detector resolution and the partial volume effect, whereby signal intensity from small lesions may be underestimated due to spatial averaging [13]. Experimental strategies, including ultrathin gadolinium-oxide nanosheets designed to enhance high-field MRI sensitivity, are under investigation; however, these remain exploratory and not part of routine clinical surveillance [13].

Temporal Lag Between Biological and Anatomical Detection

Imaging-based surveillance is also affected by an inherent temporal delay. Structural detection requires sufficient tumor growth to generate radiographically visible abnormalities. In HCC, recurrence kinetics and tumor growth patterns have been extensively characterized [14,15]; however, structural detectability remains dependent on lesion size rather than biological activity. As a result, radiographic recurrence may represent a later stage in the disease continuum. This delay may be clinically relevant in ICC, where early molecular relapse may precede anatomical confirmation.

False Positives in the Postoperative Liver

Another important limitation of imaging in ICC surveillance is the challenge of distinguishing recurrent malignancy from benign postoperative changes. Inflammation, fibrosis, and post-surgical architectural distortion may mimic tumor recurrence on CT or MRI. PET imaging may further complicate interpretation, as increased metabolic activity can occur in both inflammatory and malignant tissue.

Advanced imaging approaches such as delayed PET/MRI have demonstrated potential improvements in lesion characterization compared to PET/CT in selected settings [16]. However, diagnostic performance remains influenced by lesion size and patient-specific factors [17]. Quantitative radiomics-based approaches are being explored to improve differentiation between malignant and benign hepatic lesions, including small metastases and abscesses [18], though these techniques remain adjunctive rather than definitive.

Emerging technologies (investigational context)

Technological advancements continue to refine imaging capability. Integrated PET/MRI combines functional and anatomical data and has shown promise in detecting metabolically active hepatic malignancies [19,20]. In HCC, hepatocyte-specific contrast agents such as gadoxetate disodium have demonstrated improved lesion detection in certain settings [21]. Additionally, artificial intelligence-based tools for lesion detection and segmentation on MRI and CT are under development, with the aim of improving reproducibility and measurement accuracy [22,23]. Broader efforts to transform liver cancer detection through advanced imaging analytics are ongoing [10,11,24]. However, the clinical impact of these technologies in routine postoperative ICC surveillance remains to be fully established.

The power of liquid biopsy (ctDNA and CTCs)

Despite growing interest in integrating molecular diagnostics into liver cancer care pathways [24], ctDNA-based surveillance in biliary tract malignancies remains subject to important limitations. In biliary tract cancers, reported detection sensitivity varies across studies and depends on assay platform, sequencing depth, and tumor burden [25,26]. Broader liquid biopsy literature also highlights variability in circulating DNA release and analytical performance across tumor types [26]. Consequently, false-negative results may occur, and a negative ctDNA result cannot reliably exclude microscopic residual disease.

In addition, optimal postoperative sampling intervals have not been standardized in biliary tract cancers, and current monitoring strategies are largely extrapolated from broader solid tumor experience [25,26]. Clinically actionable thresholds such as variant allele frequency (VAF) cutoffs or criteria for confirmatory repeat testing have not been uniformly defined. Importantly, initiating systemic or liver-directed therapy solely on the basis of molecular recurrence without radiographic confirmation remains investigational. Prospective studies are required to clarify performance characteristics and determine whether ctDNA-guided intervention improves survival outcomes in biliary tract cancers.

Molecular sensitivity of ctDNA

ctDNA comprises fragmented tumor-derived DNA released into the bloodstream, carrying somatic alterations that enable molecular detection [27,28]. Detecting these minute quantities of tumor DNA within a large background of normal cell-free DNA represents a central technical challenge in ctDNA analysis [29,30]. Advanced liquid biopsy platforms, including error-corrected next-generation sequencing (NGS), enhance analytical sensitivity and allow detection at low VAFs [29,30]. Tumor-informed approaches, such as personalized assays that incorporate matched tumor and normal sequencing, are designed to improve specificity and reduce background noise [31]. Accurate interpretation also requires the discrimination of true tumor-derived variants from sequencing artifacts and mutations associated with clonal hematopoiesis of indeterminate potential, often necessitating matched leukocyte sequencing [32].

Lead time in recurrence detection

One of the potential advantages of ctDNA analysis is its ability to detect MRD before radiographic progression becomes apparent [29,33-35]. In colorectal cancer, postoperative ctDNA detection has been shown to identify patients at increased risk of recurrence following definitive treatment [34]. Across multiple tumor types, ctDNA positivity has been associated with recurrence even when imaging shows no evidence of disease [35,36]. However, the magnitude and timing of this lead interval vary across cancer types and assay platforms. In biliary tract cancers, ctDNA is being explored as a surveillance tool for MRD detection and prognostic stratification in surgically treated patients [36,37]. While early molecular detection may provide a theoretical window for earlier intervention, standardized thresholds for clinical action remain undefined. The short biological half-life of ctDNA, measured in minutes to hours, supports its role as a dynamic biomarker of active disease burden [27].

Genetic profiling and precision medicine

**Figure 1:**
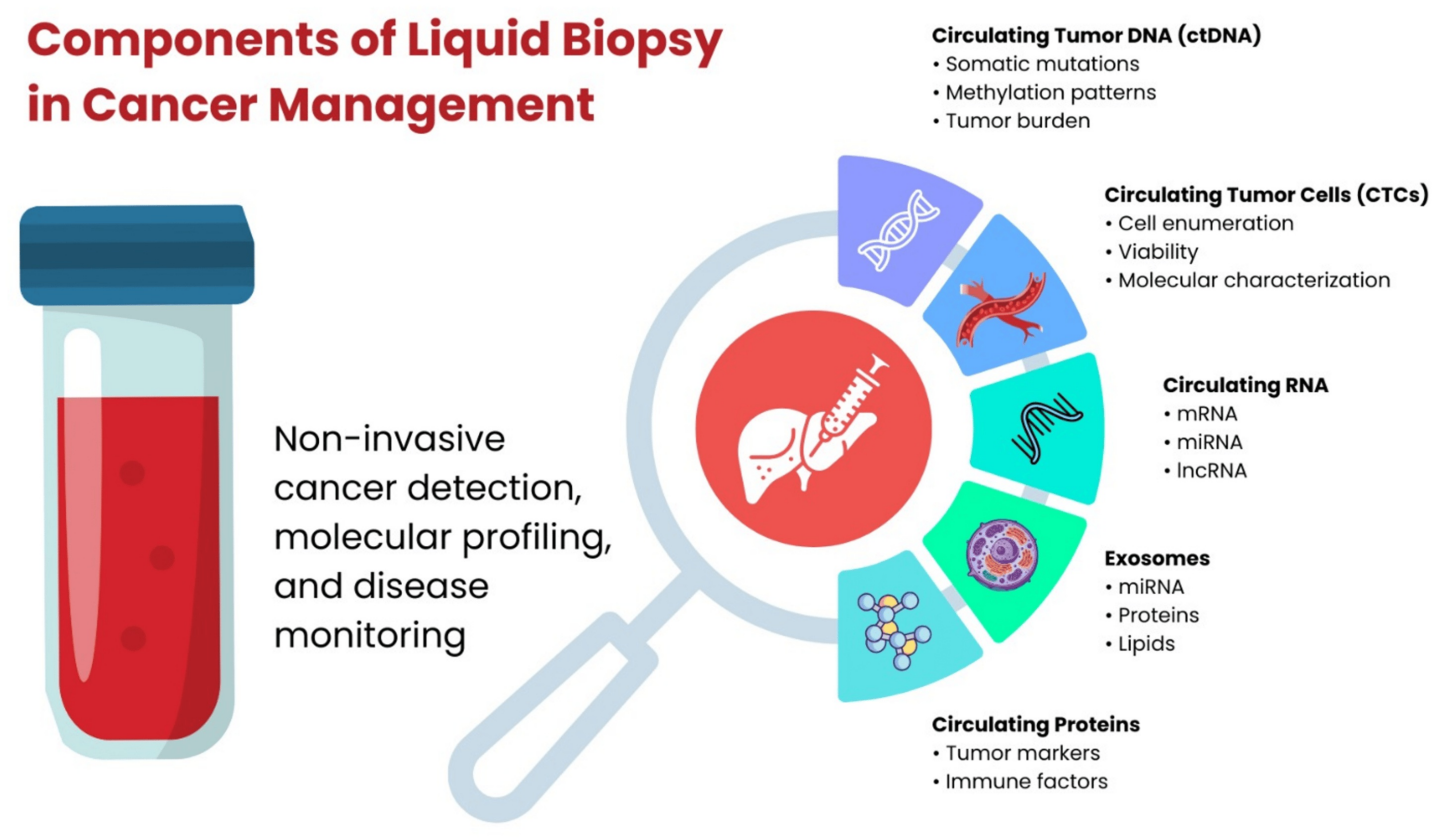
Components of liquid biopsy in cancer management The figure was created using Microsoft PowerPoint (Microsoft Corporation, Redmond, Washington, United States) for schematic illustration.

The diverse components that can be analyzed in a liquid biopsy, including ctDNA and CTCs, and their roles in cancer management are illustrated in Figure 1 [38].

Proposed hybrid surveillance framework

In light of these challenges, we propose a postoperative surveillance strategy for ICC that combines ctDNA assessment with conventional imaging. This integrated approach aims to improve the early detection of recurrence while preserving the complementary strengths of anatomical and molecular monitoring. This approach is designed to enable the early detection of molecular recurrence, risk-adapted surveillance intensity, and timely therapeutic intervention while minimizing unnecessary imaging in patients at lower risk. The proposed surveillance strategy, including the timing and rationale for each assessment, is summarized in Table 1 [8,25,35-37].

**Table 1:** Proposed hybrid surveillance strategy for postoperative monitoring in intrahepatic cholangiocarcinoma ctDNA: circulating tumor DNA; MRD: minimal residual disease; MRI: magnetic resonance imaging

Time point after surgery	Surveillance modality	Clinical rationale	Key clinical implications
Postoperative month 1	ctDNA (baseline)	Establishes molecular baseline following curative-intent resection; assesses presence of MRD in real time due to the short half-life of ctDNA	ctDNA positivity indicates persistent or disseminated disease and high recurrence risk; ctDNA negativity suggests molecular remission and favorable prognosis, enabling early risk stratification and guidance for adjuvant therapy decisions
Postoperative month 3	ctDNA + MRI	ctDNA enables the early detection of molecular recurrence after the resolution of post-surgical inflammation, often preceding radiographic recurrence; MRI provides anatomical localization and lesion characterization	Concordant ctDNA positivity and MRI findings support early intervention; discordant results prompt closer surveillance or further diagnostic evaluation, maximizing early detection through combined molecular and anatomical assessment
Postoperative month 6 (if prior ctDNA is negative)	ctDNA only	Continued molecular surveillance in patients with persistently negative ctDNA, reflecting low residual disease burden	Reduces imaging frequency, radiation exposure, and cost while maintaining high molecular sensitivity for early recurrence detection
Any time point with ctDNA positivity	Targeted imaging ± biopsy	Molecular evidence of recurrence warrants anatomical confirmation and characterization	Enables the timely localization of disease and informs treatment planning
At molecular recurrence	ctDNA-guided genomic profiling	Identification of actionable mutations through liquid biopsy	Facilitates the selection of targeted therapies (e.g., FGFR2 or IDH1 inhibitors) without the need for invasive tissue re-biopsy

## Conclusions

Follow-up after curative resection for ICC remains clinically challenging. Current standard-of-care postoperative surveillance relies primarily on interval cross-sectional imaging without the incorporation of molecular monitoring. While effective for detecting established macroscopic disease, this approach is inherently limited in identifying microscopic residual tumor and frequently detects recurrence only after radiographic visibility has occurred, at which point tumor biology may already be less favorable. Liquid biopsy offers a fundamentally different type of information. Detection of ctDNA or CTCs reflects ongoing biological tumor activity rather than delayed anatomical change. In several solid tumor settings, molecular recurrence has been observed to precede radiographic recurrence, suggesting a clinically relevant interval during which earlier intervention might be possible, particularly in patients who remain candidates for repeat resection or timely systemic therapy. At the same time, molecular surveillance has important limitations. Assay performance is not uniform, results may be influenced by biological noise and technical variability, and low-level positivity should not be acted upon in isolation. Cost considerations and tumor shedding variability further complicate implementation. For these reasons, liquid biopsy cannot currently replace imaging. A combined strategy is therefore more realistic. Molecular signals may suggest early relapse, while imaging provides anatomical confirmation and guides intervention planning. Integrating both modalities aligns postoperative monitoring more closely with tumor biology than reliance on imaging alone. Further prospective studies are required to define optimal testing intervals, determine when molecular recurrence should trigger treatment, and evaluate clinical and economic impact. Until such data are available, an integrated imaging-molecular surveillance framework represents a rational and clinically pragmatic approach for monitoring recurrence after the resection of ICC.
